# Editorial: A new frontier in translational research on autoinflammatory diseases - various aspects of innate immunity on human diseases

**DOI:** 10.3389/fimmu.2023.1147202

**Published:** 2023-01-31

**Authors:** Tomoyuki Mukai, Hiroaki Ida, Yasuyoshi Ueki, Ryuta Nishikomori

**Affiliations:** ^1^ Department of Immunology and Molecular Genetics, Kawasaki Medical School, Kurashiki, Japan; ^2^ Division of Respirology, Neurology, and Rheumatology, Department of Medicine, Kurume University School of Medicine, Kurume, Japan; ^3^ Department of Biomedical Sciences and Comprehensive Care, Indiana University School of Dentistry, Indianapolis, IN, United States; ^4^ Indiana Center for Musculoskeletal Health, Indiana University School of Medicine, Indianapolis, IN, United States; ^5^ Department of Pediatrics and Child Health, Kurume University School of Medicine, Kurume, Japan

**Keywords:** autoinflammatory disease, innate immunity, inflammasome, inflammatory cytokines, macrophage, autoinflammatory bone disease

Autoinflammatory diseases are a group of immune dysregulation disorders caused by mutations in genes related to innate immunity. Recent advances in genetic testing have led to the discovery of novel diseases. Research into the pathological mechanisms of newly identified diseases can help uncover essential inflammatory pathways. Clinicians have several options for anti-inflammatory drugs such as corticosteroids, colchicine, tumor necrosis factor (TNF)-α inhibitors, interleukin (IL)-1β inhibitors, IL-6 inhibitors, and JAK inhibitors. Although some diseases respond well to existing treatments, others currently have no established treatments. A reciprocal approach that involves testing the effectiveness of existing treatments and developing new ones is needed to treat patients better. This Research Topic gathered eight articles that could improve our understanding of the pathogenesis and management of autoinflammatory diseases.

Blau syndrome is a systemic autoinflammatory granulomatous disease caused by mutations in the *NOD2* gene. NOD2 is an intracellular pathogen recognition receptor that binds to the muramyl dipeptide, a component of the bacterial cell wall. Matsuda et al. summarize the pathogenesis of Blau syndrome and the efficacy of TNF-α-targeting therapy. They recently reported that TNF-α pretreatment of monocytes with Blau syndrome-associated *NOD2* mutations augments inflammatory responses to interferon (IFN)-γ (1). Building on these findings, they address the effectiveness of TNF-α inhibitors, presumably mediated by the reduction in the TNF-α-mediated priming effect on monocytes with *NOD2* mutations.

Type I interferonopathies are a subgroup of autoinflammatory diseases characterized by dysregulation of the IFN pathway. Interferonopathies are caused by mutations in genes associated with proteasomal degradation or cytoplasmic DNA- and RNA-sensing pathways. As interferonopathies are clinically heterogeneous, the extent to which type I IFN is involved in the pathogenesis of each inflammatory disorder or patient remains unclear. To clarify this point, Miyamoto et al. quantified the type I IFN signature in undifferentiated inflammatory diseases. They found that half of the undiagnosed patients showed increased IFN signaling and that half of the IFN-high patients exhibited clinical features similar to interferonopathies. After detailed genetic analysis, they identified novel mutations in genes linked to type I IFN signaling in some IFN-high patients. Based on these findings, they propose that IFN signature measurement can help narrow down the candidate variants identified through genetic analysis. They also address the possibility of measuring IFN signatures for personalized medicine. From this perspective, they propose that treatment with JAK inhibitors would be beneficial for patients with high IFN signatures, even when their pathogenic mutations are not confirmed.

IL-18 is a pleiotropic proinflammatory cytokine involved in the regulation of innate and adaptive immune responses. Pro-IL-18 is constitutively expressed and ready to be processed and released by inflammatory stimuli. After inflammasome activation, caspase-1 processes pro-IL-18 as well as pro-IL-1β. Some inflammasome components induce high levels of IL-18 production, leading to the development of IL-18-dominant autoinflammation and macrophage activation syndrome (MAS). Shimizu et al. and Miyazawa and Wada summarize the current understanding of IL-18-dominant autoinflammation. Shimizu et al. describe the implications of IL-18 in the development of MAS, including systemic juvenile idiopathic arthritis and adult-onset Still’s disease, and propose the diagnostic utility of IL-18 in autoinflammatory diseases. Miyazawa and Wada present the pathogenic mechanisms of IL-18 in X-linked inhibitor of apoptosis deficiency, NLRC4-associated autoinflammatory disease, cell division cycle 42 C-terminal disease, PSTPIP1-associated inflammatory diseases, and WD repeat-containing domain 1 deficiency. Both review articles demonstrate that chronic excess of serum IL-18 is linked to predisposition to MAS; both studies also emphasize that excess of IL-18 is not the single inducer for MAS, referring to the fact that PSTPIP1-associated inflammatory diseases are less likely to develop MAS. Further analysis is required to determine the multifactorial pathways leading to hyper-IL-18 and the development of MAS, which would aid in establishing IL-18-targeted therapy for autoinflammation and MAS.

IL-6 is not directly associated with inflammasomes but increases in the inflammatory milieu in autoinflammatory diseases. Koga and Kawakami review the role of IL-6 in autoinflammatory diseases and summarize studies on IL-6 inhibition in autoinflammatory diseases, including familial Mediterranean fever (FMF) and Bechet disease. They previously conducted a clinical trial to evaluate the efficacy and safety of tocilizumab in patients with FMF (2). Although the primary goal of reducing the number of fever attacks was not achieved, tocilizumab effectively reduced febrile attacks and other symptoms. They propose that an anti-IL-6 receptor antibody, tocilizumab, is recommended for patients with FMF in the early stages of AA amyloidosis and for those unresponsive to colchicine to prevent the development of AA amyloidosis.

Vacuoles, E1 enzyme, X-linked, autoinflammatory, somatic (VEXAS) syndrome is an adult-onset autoinflammatory disease caused by somatic mutations in the *UBA1* gene (3). Unlike other genetic autoinflammatory diseases, which in most cases are caused by germline mutations, VEXAS syndrome is induced by acquired variants in hematopoietic cells, especially myeloid progenitors. VEXAS syndrome causes inflammatory and hematological manifestations. The lungs, joints, skin, eyes, and gastrointestinal system are frequently affected. In addition, half of the patients exhibit hematological involvement, mainly myelodysplastic syndrome. Currently, there is no established treatment for VEXAS syndrome. Kunishita et al. present the one-year efficacy of tocilizumab in three patients with VEXAS syndrome. Tocilizumab treatment suppressed inflammatory manifestations but not hematological abnormalities. They propose that tocilizumab is effective for inflammation-dominant VEXAS syndrome and could serve as a bridging therapy for future treatment development.

Autoinflammatory bone diseases are characterized by sterile bone inflammation and recognized as a branch of autoinflammatory diseases (4). They include chronic recurrent multifocal osteomyelitis (CRMO), Majeed syndrome, deficiency of IL-1 receptor antagonist, and cherubism. Sergi et al. summarize the current understanding of CRMO, focusing mainly on its clinical aspects. Various bone lesions are presented in CRMO, including the metaphysis of the long bones, pelvis, spine, clavicle, and mandible. The benefit of whole-body magnetic resonance imaging as a noninvasive diagnostic tool is emphasized because the bone lesions of CRMO are often multifocal and subclinical.

TNF receptor-associated periodic syndrome (TRAPS) is a prototype autoinflammatory disease TRAPS is caused by mutations in *TNFRSF1A*, which encodes tumor necrosis factor receptor 1 (TNFR1). Although TRAPS was first reported in 1999 (5), its pathogenesis is still not fully understood. The most likely hypothesis is that mutated TNFR1 accumulates in the endoplasmic reticulum (ER), and consequent ER stress enhances susceptibility to inflammatory stimuli (6). Akagi et al. generated three strains of TRAPS mutant mice by introducing three TRAPS-associated mutations into murine *Tnfrsf1a*. They showed that TRAPS mutant mice are unexpectedly resistant to lipopolysaccharide and TNF-α, which is associated with decreased cell surface expression of TNFR1. Increased ER stress responses were not observed with the tested stimuli. They considered the presence of disease-specific inflammation-inducing factors; however, details of the pathology remain unclear.

These excellent reports cover a broad spectrum of research in autoinflammatory diseases. We hope that this Research Topic will improve our understanding of the pathogenesis of each condition and provide a better foundation for the application and future development of treatments.

## Author contributions

All authors listed have made a substantial, direct, and intellectual contribution to the work and approved it for publication.

## References

[1] Kitagawa Y Kawasaki Y Yamasaki Y Kambe N Takei S Saito MK. Anti-TNF treatment corrects IFN-γ–dependent proinflammatory signatures in blau syndrome patient–derived macrophages. J Allergy Clin Immunol (2022) 149:176–188. e7. doi: 10.1016/j.jaci.2021.05.030 34175136

[2] Koga T Sato S Hagimori N Yamamoto H Ishimura M Yasumi T. A randomized, double-blind, placebo-controlled phase III trial on the efficacy and safety of tocilizumab in patients with familial Mediterranean fever. Clin Exp Rheumatol (2022) 40(8):1535–42. doi: 10.55563/clinexprheumatol/fgx9vv 36106542

[3] Beck DB Ferrada MA Sikora KA Ombrello AK Collins JC Pei W. Somatic mutations in UBA1 and severe adult-onset autoinflammatory disease. New Engl J Med (2020) 383:2628–38. doi: 10.1056/NEJMoa2026834 PMC7847551 33108101

[4] Morbach H Hedrich CM Beer M Girschick HJ. Autoinflammatory bone disorders. Clin Immunol (2013) 147:185–96. doi: 10.1016/j.clim.2012.12.012 23369460

[5] McDermott MF Aksentijevich I Galon J McDermott EM Ogunkolade BW Centola M. Germline mutations in the extracellular domains of the 55 kDa TNF receptor, TNFR1, define a family of dominantly inherited autoinflammatory syndromes. Cell (1999) 97:133–44. doi: 10.1016/S0092-8674(00)80721-7 10199409

[6] Simon A Park H Maddipati R Lobito AA Bulua AC Jackson AJ. Concerted action of wild-type and mutant TNF receptors enhances inflammation in TNF receptor 1-associated periodic fever syndrome. Proc Natl Acad Sci (2010) 107:9801–6. doi: 10.1073/pnas.0914118107 PMC2906866 20457915

